# Multi-domain biopsychosocial postoperative recovery trajectories associate with patient outcomes following lumbar fusion

**DOI:** 10.1007/s00586-023-07572-0

**Published:** 2023-03-06

**Authors:** Ryan T. Halvorson, Abel Torres-Espin, Matthew Callahan, Bobby Tay, Conor O’Neill, Sigurd Berven, Jeffrey C. Lotz, Jeannie F. Bailey

**Affiliations:** 1 Department of Orthopedic Surgery, University of California, San Francisco, USA; 2 Department of Neurological Surgery, University of California, San Francisco, USA; 3 Department of Physical Therapy, University of Alberta, Edmonton, Canada

**Keywords:** Lumbar spine, Spinal fusion, Recovery trajectories, Biopsychosocial, Patient outcomes

## Abstract

**Purpose:**

The purpose of this study is to describe and assess the impact of multi-domain biopsychosocial (BPS) recovery on outcomes following lumbar spine fusion. We hypothesized that discrete patterns of BPS recovery (e.g., clusters) would be identified, and then associated with postoperative outcomes and preoperative patient data.

**Methods:**

Patient-reported outcomes for pain, disability, depression, anxiety, fatigue, and social roles were collected at multiple timepoints for patients undergoing lumbar fusion between baseline and one year. Multivariable latent class mixed models assessed composite recovery as a function of (1) pain, (2) pain and disability, and (3) pain, disability, and additional BPS factors. Patients were assigned to clusters based on their composite recovery trajectories over time.

**Results:**

Using all BPS outcomes from 510 patients undergoing lumbar fusion, three multi-domain postoperative recovery clusters were identified: Gradual BPS Responders (11%), Rapid BPS Responders (36%), and Rebound Responders (53%). Modeling recovery from pain alone or pain and disability alone failed to generate meaningful or distinct recovery clusters. BPS recovery clusters were associated with number of levels fused and preoperative opioid use. Postoperative opioid use (*p* < 0.01) and hospital length of stay (*p* < 0.01) were associated with BPS recovery clusters even after adjusting for confounding factors.

**Conclusion:**

This study describes distinct clusters of recovery following lumbar spine fusion derived from multiple BPS factors, which are related to patient-specific preoperative factors and postoperative outcomes. Understanding postoperative recovery trajectories across multiple health domains will advance our understanding of how BPS factors interact with surgical outcomes and could inform personalized care plans.

## Introduction

Spinal disorders impact patients across multiple biopsychosocial (BPS) health domains, including diverse aspects of mental and physical wellbeing. Poor outcomes following spine surgery are relatively frequent compared to other orthopedic surgical procedures, and can contribute to long-term opioid consumption, emergency department (ED) utilization, readmissions and reoperations [1, 2]. BPS factors are an important comorbidity that may have a measurable impact on patient-reported health status, and on the outcomes of care [3]. Furthermore, BPS factors may be a modifiable risk factor for poor outcomes and their role on recovery is a critical and overlooked avenue for improving spinal surgery outcomes. Forecasting patient response to interventions preoperatively could guide healthcare providers to intervene with precise, patient-specific treatment plans. The majority of previous work using BPS factors to predict risk for postoperative outcomes only considers baseline BPS data [3, 4]. Prior studies generally do not consider multi-domain heterogeneity in patient responses and do not capture the separate yet connected effects of different BPS factors on overall longitudinal response trajectories following surgery. The purpose of this study is to describe and assess the impact of multi-domain BPS recovery on outcomes following lumbar spine fusion.

**Fig. 1:**
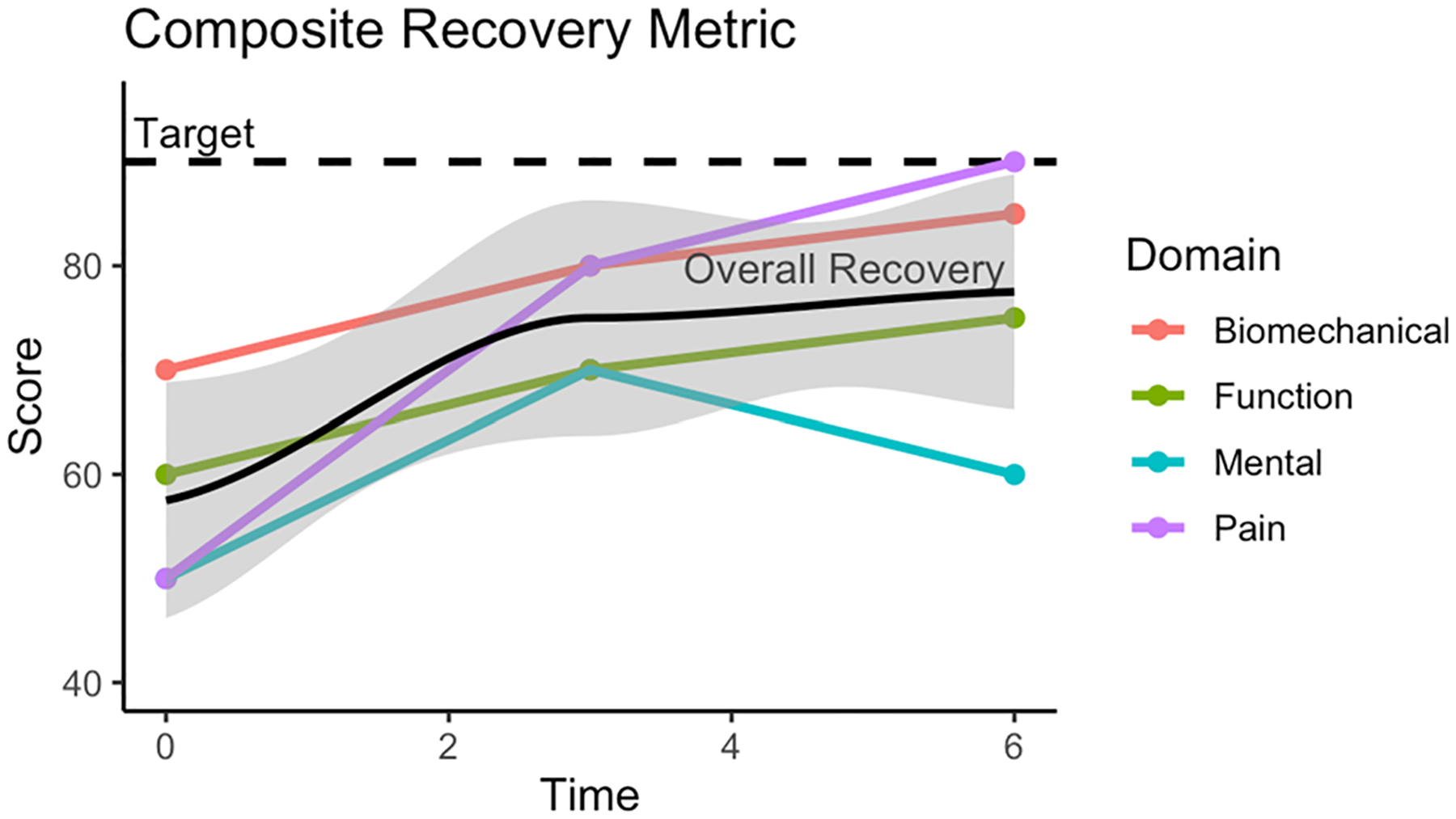
Schematic depicting multi-domain patient composite recovery trajectory. This figure depicts theoretical patient recovery following a surgical intervention. The patient responds at different rates and patterns with respect to biomechanics, function, mental health, and pain. Each of these example domains is represented with a different colored line. In black is the patient’s overall “Composite Recovery”, which quantifies overall recovery as a function of each individual domain

Understanding a patient’s non-linear response trajectory following spine surgery grants insight into the rates and patterns of recovery that are otherwise not captured in basic outcomes analyses comparing direct changes between two timepoints (preoperative and postoperative). Information regarding the dynamic behavior of recovery after spine surgery is valuable in guiding expectations for surgery and in developing early recovery programs that are responsive to the needs of specific patients [4]. Classifying a patient’s response to surgery based on unique trajectories of recovery may guide expectations regarding long-term outcomes and risk of postoperative complications [5–8]. The PROMIS is designed to measure patient-reported health status in multiple domains including pain, fatigue, physical function, emotional distress, and social role [9]. The recovery trajectory for patients may vary between these domains, and information regarding patient-specific recovery trajectories is useful to guide patient expectations, and to predict outcomes in different domains of health status. Multi-domain recovery trajectories built from longitudinal patient-reported outcomes (Fig. 1) may reveal novel and unique relationships with objective clinical outcomes and overall long-term recovery following treatment.

To capture patient-specific trajectories of postoperative recovery and account for multiple patient BPS outcome domains, we collected multi-domain patient-reported outcomes data on spine surgery patients at multiple time points spanning from baseline to one-year follow-up. We hypothesized that separate multi-domain trajectory clusters for response to treatment would (1) associate with postoperative clinical outcomes (prolonged opioid use, hospital length of stay (LOS)), and (2) be predicted by baseline demographic and clinical information. The ability to preoperatively anticipate a patient’s rate of change and overall improvement during recovery will empower informed choice regarding surgery. Understanding how BPS factors may contribute to expected outcomes will provide opportunity for preoperative optimization of modifiable factors to optimize outcomes of care.

## Methods

### Subjects, study design, and outcomes

This observational longitudinal cohort study is approved by our institution’s ethics board. Adult patients undergoing spinal fusion involving the lumbar spine at an academic institution between 2019 and 2022 were eligible for inclusion. Patients were excluded who had isolated surgery of the thoracic or cervical spine, or failed to complete at least two patient-reported outcome surveys. Outcomes collected included Owestry Disability Index (ODI) and PROMIS Global 10 2.9v domains: Anxiety, Depression, Fatigue, Pain Interference, and Social Roles (Table 1). Data were recorded at approximately baseline (preoperative), three months, six months, and one year after fusion. All scores for each PROMIS domain and ODI range 0–100 with higher scores representing worse symptoms.

Baseline demographic factors (age, sex, BMI, race, ethnicity), medical factors (American Society of Anesthesiologist Classification, Charleston Comorbidity Index, preoperative opioid use), and surgical factors (number of levels, spine region, surgical approach) were recorded, as well as hospital LOS, postoperative opioid use, readmissions within 30 days, and ED visits within 30 days. Postoperative opioid use was quantified using oral morphine equivalents.

### Composite recovery models

To assess composite recovery as a function of multiple diverse PROs, multivariable latent class mixed models were employed [10]. To assess for the impact of additional BPS variables versus traditional single outcome modeling, three models were generated sequentially to cluster observed response trajectories: one using pain alone (pain interference), one using pain and disability (ODI), and one using pain, disability, and a full set of BPS variables derived from the PROMIS 2.9v domains (pain, anxiety, fatigue, depression, and social roles). This technique assumes each PRO is a biomarker of an underlying latent process, which represents the patient’s experience of recovery following surgery. Each response variable is mapped onto the latent variable using a prespecified link function. Models were generated by specifying two to four trajectory clusters and optimized according to Bayesian and Akaike information criteria [11], and minimum cluster size of 5%. Time was modeled as a continuous variable using the exact dates of survey completion, and non-linear trajectories were modeled using a b-spline basis with 4 degrees of freedom. Patients were assigned to the trajectory cluster with higher posterior probability of membership.

### Statistical analyses

Associations between trajectory cluster membership and baseline demographic, medical, and surgical factors were first assessed using univariable analyses, including ANOVA for continuous variables and Chi- squared tests for categorical variables. Predictors significant at the 0.10 level were included in a multivariable multinomial logistic regression to predict patient cluster as the response variable.

Associations between postoperative outcomes (LOS, postoperative opioid use, readmissions, and ED visits) were assessed using univariable analysis. Multivariable linear regression was employed to assess for the impact of patient cluster on postoperative opioid utilization and hospital LOS, while controlling for other baseline medical and surgical information (sex, age, BMI, preoperative opioid use, ASA class, and number of levels fused). All analyses are carried out in R [12].

## Results

### Sample characteristics

**Fig. 2:**
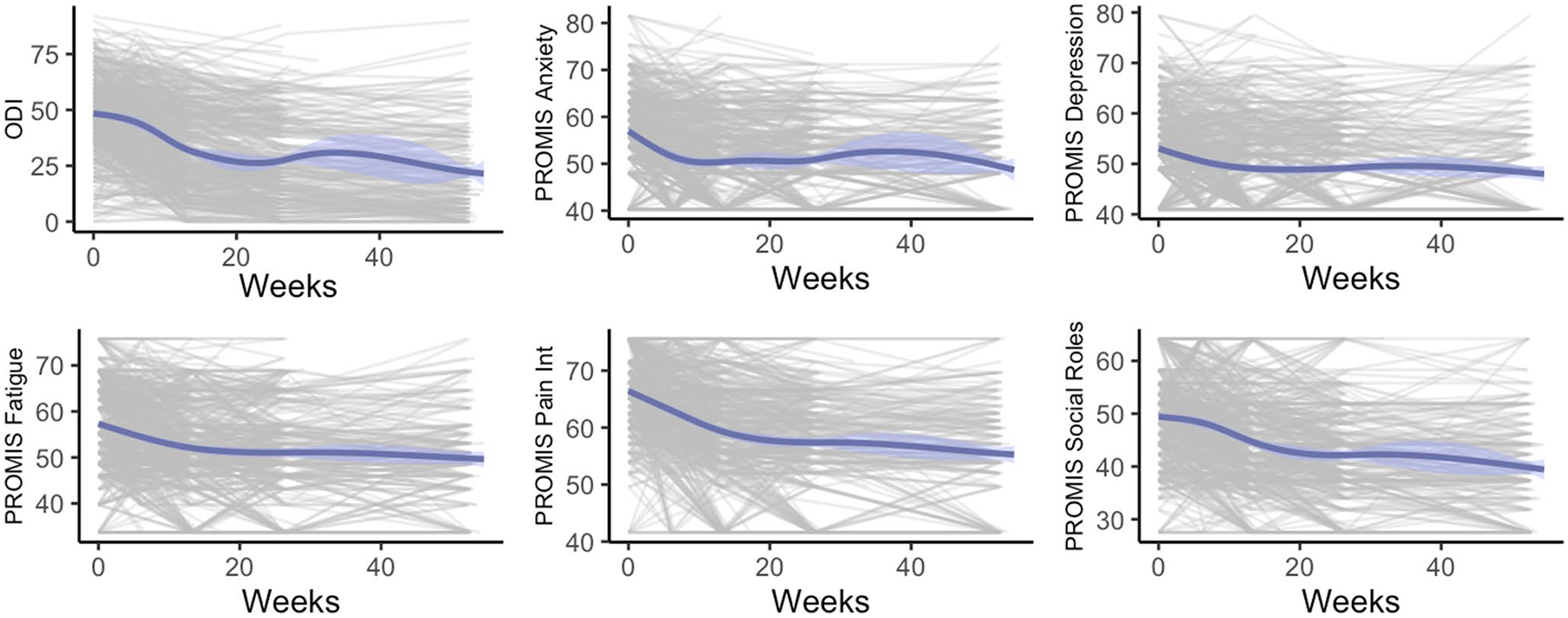
Observed postoperative trajectories for ODI, pain, and PROMIS sub-scores. Each plot depicts raw recovery trajectories in each domain for each patient included in the cohort in grey. Each line represents the longitudinal outcome from a single patient. The blue lines and 95% confidence intervals depict the univariate average trajectory across all subjects

709 patients met inclusion criteria, of whom 510 (72.0%) completed surveys at multiple time points and were included in the final clustering models (Fig. 2). The cohort was 53.1% female with mean age 65.0. 164 patients underwent fusion of 1–2 levels, 177 patients 3–5 levels, 117 patients 6–11 levels, and 52 patients with more than 12 levels. Mean follow-up was 566 days (SD 344).

### Trajectory models

**Fig. 3:**
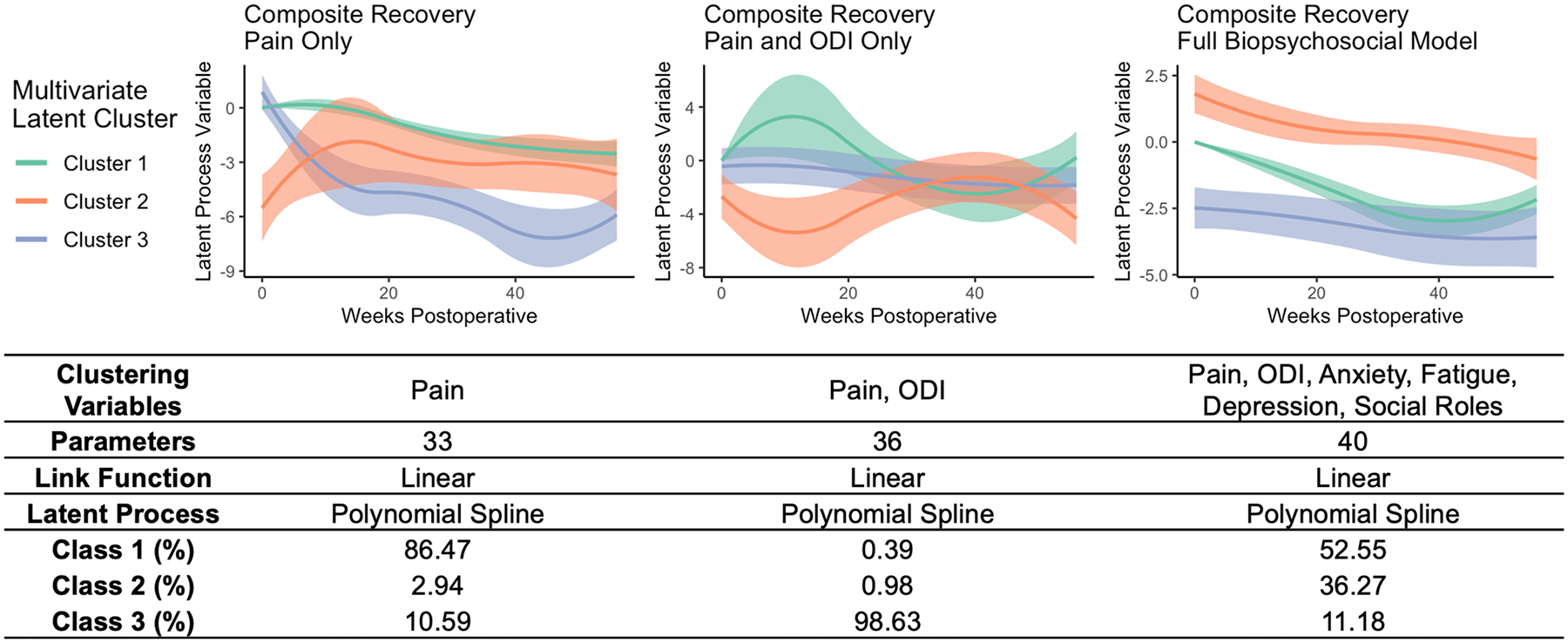
Postoperative latent trajectories for ODI, pain, and PROMIS sub-scores. Three models were generated to quantify composite recovery: one using pain alone (PROMIS Pain Interference sub-score) [left], one using pain and disability (ODI) together [center], and one using pain, disability, and a full set of BPS variables derived from the PROMIS 2.9v domains (pain, anxiety, fatigue, depression, and social roles) [right]. Graphs depict the composite recovery score as a function of time. Lines represent the mean and 95% confidence intervals for patients in each cluster identified using each model (Cluster 1: green, Cluster 2: red, Cluster 3: blue). All models were generated using a linear link function and non-linear b-spline regression to model the outcome. Cluster sizes are tabulated below for each model

The three separate models were generated sequentially using pain, disability, and BPS variables and optimized for number of clusters, which resulted in three clusters per model. The pain model was characterized by cluster sizes of 86%, 11%, and 3% with poorly distinguished latent variable trajectories. The pain and disability model similarly demonstrated cluster sizes of 98.6%, 0.4%, and 1% with poorly distinguished trajectories. Finally, the pain, disability, and BPS model resulted in cluster sizes of 53%, 11%, and 36% with distinct trajectories (Fig. 3).

In the final model, composite recovery is modeled as a latent process, with patient-reported pain, disability, anxiety, fatigue, depression and social roles serving as dynamic markers of the latent process. Similar to the markers of recovery, larger latent process values correspond to worse health. The majority of patients in this model (53%) were found to improve from a composite recovery of 0.0 points to − 2.97 points over the first year, with a rebound to − 2.16 points toward the end of the year (net 2.16 point improvement) and are thus termed Rebound Responders. A second subset representing 36.27% of patients was found to start with the worst symptoms (composite 1.8 points) and improve more moderately to − 0.64 points (2.4 point improvement) and are thus termed Rapid BPS Responders. A third subset representing 11.18% of patients was found to start with more mild symptoms (composite − 2.4 points) and also improve moderately to − 3.64 points (net 1.24 point improvement), and are thus termed Gradual BPS Responders.

### Associations with baseline patient factors and postoperative trajectories

In univariable models, sex (*p* < 0.05), ethnicity (*p* < 0.1), preoperative opioid use (*p* < 0.001), ASA class (*p* < 0.1), preoperative ODI (*p* < 0.005), preoperative pain (*p* < 0.005), number of levels fused (*p* < 0.005), and surgical approach (*p* < 0.01) were associated with BPS recovery clusters and were included in a multivariable, multinomial logistic regression model (Table 2). In the final multivariable model, male sex (males had 2.38 odds of being a Rapid BPS Responder versus a Gradual BPS Responder [95% CI 1.21–4.67, *p* = 0.01]), having a larger number of levels fused (patients having 6–11 levels fused had 0.34 odds of being a Rapid BPS Responder versus a Gradual BPS Responder [95% CI 0.12–0.96, *p* = 0.04]), and prior opioid use (patients using opioids preoperatively had a 3.53 odds of being a Rebound Responder [95% CI 1.73–7.19, *p* < 0.001] and a 7.77 odds of being a Rapid BPS Responder [95% CI 3.69–16.36, *p* < 0.001] versus Gradual BPS Responders, Table 3) were associated with BPS recovery clusters. There were no between cluster associations in ASA classification, surgical approach, or BMI.

With regards to long-term clinical outcomes, BPS recovery trajectory clusters were significantly associated with postoperative opioid use (Rebound Responders: 680 OME, Gradual BPS Responders: 1008 OME, and Rapid BPS Responders 416 OME, *p* < 0.001) and hospital LOS (Rebound Responders: 149 h, Gradual BPS Responders: 179 h, and Rapid BPS Responders 101.4 h, *p* < 0.05; Table 4). However, there was no difference in 30-day ED visits or readmissions. In multivariable linear regression models controlling for sex, age, BMI, preoperative opioid use, ASA class, and number of levels fused, BPS recovery clusters predicted both postoperative opioid utilization (*p* < 0.01) and hospital LOS (*p* < 0.01).

## Discussion

To better understand how patients experience spinal disorders and benefit from interventions, it is necessary to develop quantitative methods to assess the complex interactions of multiple relevant demographic, surgical, social, and psychological factors. From a longitudinal cohort study following postoperative recovery of 510 patients undergoing lumbar spine fusion, we identified and characterized three multi-domain postoperative recovery clusters from a diverse set of BPS outcomes. Modeling recovery using pain alone or pain and disability alone failed to generate meaningful or distinct recovery clusters; however, by incorporating BPS factors in our latent class trajectory model, we uncovered distinct recovery trajectory clusters that represented Gradual BPS Responders (11% of patients), Rapid BPS Responders (36%), and Rebound Responders (53%). The resulting BPS recovery trajectory clusters were found to be related to patient sex, number of levels fused, and preoperative opioid use in a multinomial logistic regression model. Postoperative opioid use and hospital LOS were also associated with BPS recovery trajectory clusters even after adjusting for preoperative, demographic, and surgical factors.

Our results indicate that BPS outcomes may better inform spinal surgery recovery models. A prior analysis of patients with thoracolumbar disease demonstrated that ODI alone fails to explain up to 50% of variability in PROMIS domains [13]. While pain and disability may be primary complaints leading a patient to seek treatment, the persistence of chronic pain can influence patient mental health, social roles, and pain perception [14]. These interactions between disease-related symptoms and overall health are complex, and justify the need for modeling approaches that independently consider various aspects of recovery simultaneously.

**Fig. 4:**
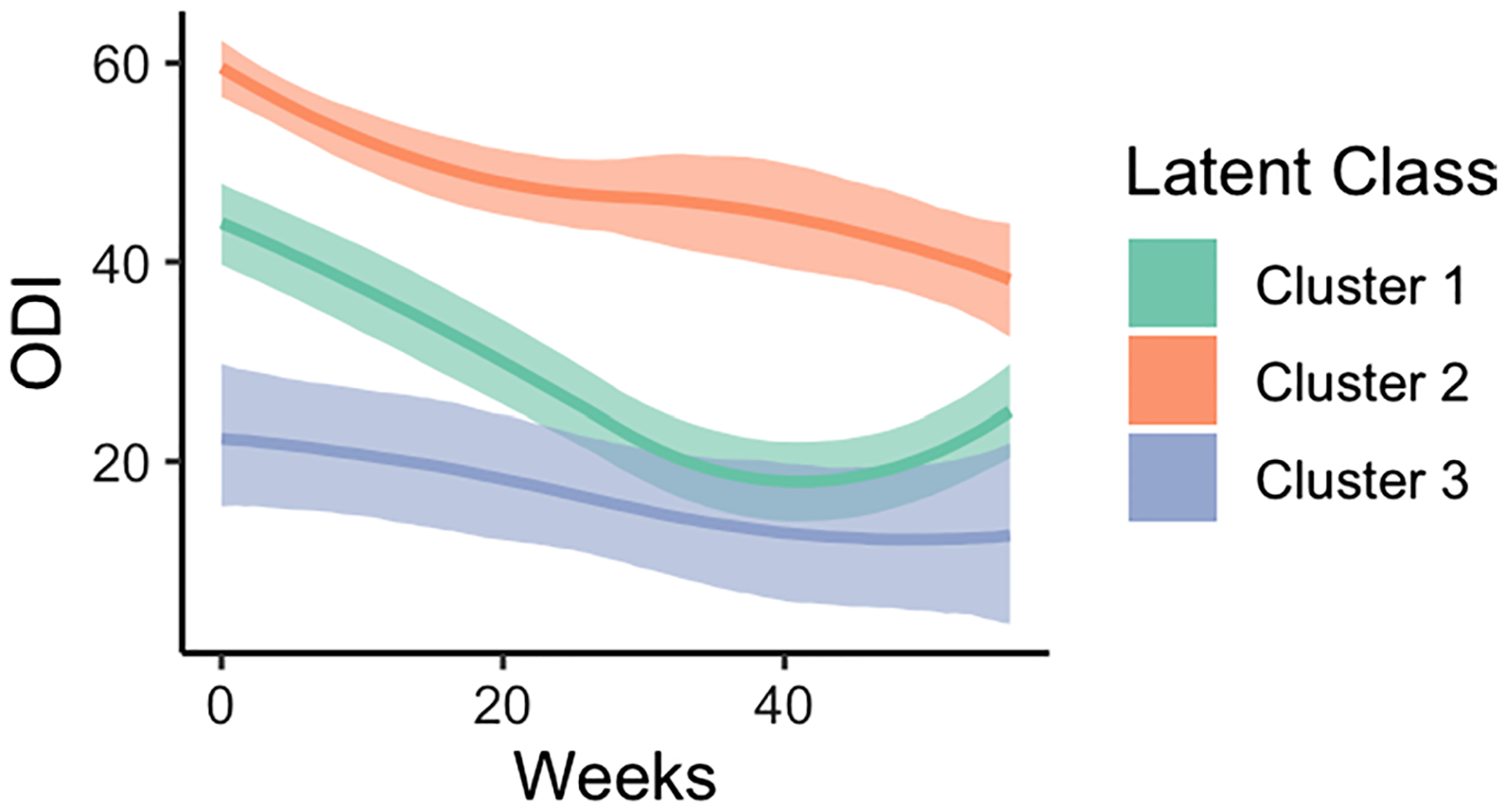
Predicted ODI from composed recovery variable derived from BPS response trajectories. Graph below depicts predicted ODI as a function of time following surgery, derived from the BPS response trajectories. Lines represent the mean and 95% confidence intervals for each cluster (Cluster 1: green, Cluster 2: red, Cluster 3: blue). All models were generated using a linear link function and non-linear b-spline regression to model the outcome. The trajectories appear similar to the composed recovery variable because a linear link function is employed

Latent process modeling can be used to reconstitute specific underlying patient-outcome trajectories for patients, which is a potentially valuable clinical tool to understand relative recovery differences for a specific outcome domain (Fig. 4). Dynamic behaviors in recovery can inform: (1) the anticipated overall improvement between trajectory clusters (1.24 points for Gradual BPS Responders, 2.4 points for Rapid BPS Responders, and 2.16 points for Rebound Responders), (2) the rate of change between trajectory clusters (gradual versus rapid responders), and (3) whether there are any changes in the rate of recovery (unexpected 0.8 point decrease in recovery for rebound responders beginning at week 40). The change in recovery for the Rebound Responders would not be captured by analyzing recovery between baseline and one-year follow-up alone and this cluster may be at heightened risk for additional treatment or a revision. Future work will more closely examine associations with incidence of readmissions and reoperations.

BPS recovery clusters were associated with postoperative outcomes, such as hospital LOS and prolonged opioid use. It is well established from prior work that that baseline factors, including preoperative opioid use, strongly predict postoperative opioid use [15]. In our analysis, a significant association between BPS recovery cluster and postoperative opioid use was still observed after controlling for preoperative opioid use in a multivariable, multinomial analysis. Outcomes such as opioid use and hospital LOS may have a particular relevance to patient-reported health perceptions. Examining differences in recovery response of underlying BPS factors like anxiety and depression between overall recovery trajectory clusters can help us create patient-specific care plans for managing these factors in effort to improve their recovery. Adding personalized multidisciplinary BPS components to postoperative rehabilitation could improve a patient’s recovery and postoperative outcomes [16]. Future work will explore how to better predict how these separate mental health and social well-being factors contribute to a patient’s anticipated recovery trajectory, as well as how these BPS recovery clusters associate with long-term outcomes.

A variety of baseline patient factors, such as patient demographics, preoperative opioid use and surgery-related details, significantly associated with the BPS recovery trajectory clusters. Much of the literature on predicting spine surgery outcomes demonstrates associations between baseline patient data and surgical outcomes, identifying potential risk factors [17]. However, these associations between baseline patient data and surgical outcomes do not capture the dynamic behaviors of overall recovery that we are characterizing with our trajectory clusters. Being able to anticipate a patient’s rate of change and overall improvement during recovery, as well as how underlying BPS factors contribute, provides more actionable forecasts for postoperative response and creating patient-specific care plans for improving outcomes.

By simultaneously modeling multiple distinct domains of patient recovery, this analysis represents a novel and pragmatic approach to postoperative outcomes following lumbar spine surgery. However, there are several key limitations. First, there may be a larger number of clinical recovery phenotypes that could not be quantified in this analysis due to sample size. Because some complications, including revisions, continue to occur in the long-term (e.g., after several years), this study may not capture the complication rate for this cohort, and was underpowered to assess for revisions. Importantly, this study does not adjust for postoperative treatment (e.g., compliance with physical therapy), which may affect recovery. Finally, this study was limited to a single academic institution and may be subject to regional differences in outcomes.

The patient experience of spinal disorders and related chronic pain is complex; pain and disability are important parameters in recovery, but are influenced by multiple diverse medical, surgical, social, and psychological pathways. Modern quantitative methods have enabled the collection and analysis of diverse data at higher sampling frequencies than was previously possible. This study suggests that there may be exist distinct multi-domain BPS recovery trajectories following lumbar spine fusion, which are related to preoperative factors and postoperative outcomes, and may be used to inform personalized care plans.

## Figures and Tables

**Table 1:** Survey items underlying biopsychosocial variables

BPS variable	Survey item	Scoring
Depression	How often have you been bothered by emotional problems such as feeling depressed?	Never; Rarely; Sometimes; Often; Always
Anxiety	How often have you been bothered by emotional problems such as feeling anxious?	Never; Rarely; Sometimes; Often; Always
Fatigue	How would you rate your fatigue on average?	None; Mild; Moderate; Severe; Very Severe
Social Roles	In general, please rate how well you carry out your usual social activities and roles. (This includes activities at home, at work and in your community, and responsibilities as a parent, child, spouse, employee, friend, etc.)	Poor; Fair; Good; Very Good; Excellent

**Table 2:** Associations between BPS trajectories and preoperative demographic, medical, and surgical factors

	Cluster 1: rebound responders	Cluster 2: rapid BPS responders	Cluster 3: gradual BPS responders	*P* value
Number	258	176	57	
Sex				**0.017**
Female	142 (52.99%)	108 (58.38%)	21 (36.84%)	
Age				0.39
Over 75	54 (20.15%)	30 (16.22%)	8 (14.04%)	
Under 75	214 (79.85%)	155 (83.78%)	49 (85.96%)	
Ethnicity				**0.058**
Non-Hispanic	247 (92.16%)	172 (92.97%)	54 (94.74%)	
Hispanic	19 (7.09%)	13 (7.03%)	1 (1.75%)	
Race				0.24
White	226 (84.33%)	149 (80.54%)	46 (80.7%)	
Black	10 (3.73%)	5 (2.7%)	2 (3.51%)	
Asian	10 (3.73%)	12 (6.49%)	7 (12.28%)	
Other	21 (7.84%)	19 (10.27%)	2 (3.51%)	
Preoperative opioid use				** < 0.001**
Yes	128 (47.76%)	125 (67.57%)	13 (22.81%)	
No	140 (52.24%)	60 (32.43%)	44 (77.19%)	
BMI				**0.067**
Normal	93 (34.7%)	60 (32.43%)	24 (42.11%)	
Overweight	100 (37.31%)	57 (30.81%)	18 (31.58%)	
Obese	72 (26.87%)	68 (36.76%)	15 (26.32%)	
ASA Class				**0.051**
1–2	179 (66.79%)	105 (56.76%)	41 (71.93%)	
3–4	89 (33.21%)	78 (42.16%)	16 (28.07%)	
Not Recorded	0 (0%)	2 (1.08%)	0 (0%)	
CCI (SD)	3.0 (1.9)	3.1 (2.2)	2.8 (1.7)	0.41
Preoperative PRO				
ODI (SD)	43.5 (11.9)	61.5 (11.5)	17.9 (11.2)	** < 0.001**
Pain (SD)	65.0 (4.8)	71.7 (4.1)	53.1 (5.9)	** < 0.001**
Levels fused				**0.002**
1–2	86 (32.09%)	46 (24.86%)	32 (56.14%)	
3–5	91 (33.96%)	70 (37.84%)	16 (28.07%)	
6–11	61 (22.76%)	50 (27.03%)	6 (10.53%)	
12 or more	30 (11.19%)	19 (10.27%)	3 (5.26%)	
Approach				**0.057**
Posterior	104 (38.81%)	75 (40.54%)	32 (56.14%)	
Anterior	14 (5.22%)	4 (2.16%)	3 (5.26%)	
Circumferential	150 (55.97%)	106 (57.3%)	22 (38.6%)	
Region				1
Lumbar-Sacral	24 (8.96%)	28 (15.14%)	3 (5.26%)	
Lumbar	152 (56.72%)	86 (46.49%)	45 (78.95%)	
Thoracic-Lumbar	17 (6.34%)	17 (9.19%)	1 (1.75%)	
Thoracic-Sacral	75 (27.99%)	54 (29.19%)	8 (14.04%)	
Case time in hours (SD)	5.13 (2.6)	5.11 (2.3)	4.91 (2.4)	0.89

Associations between trajectory BPS cluster membership and baseline demographic, medical, and surgical factors are tabulated according to either mean or standard deviation for continuous variables or count and percentage for categorical variables. *P* values represent the results of univariable analyses, including ANOVA for continuous variables and Chi-squared tests for categorical variables

**Table 3:** Multinomial regression to predict response cluster from preoperative demographic, medical, and surgical factors

	Cluster 3: gradual BPS responders	Cluster 1: rebound responders	*P* value	Cluster 2: rapid BPS responders	*P* value
Number of levels					
1 to 2		Reference		Reference	
3 to 5	Reference	1.67 (0.78–3.54)	0.18	0.45 (0.2–1.02)	0.06
6 to 11	Reference	2.38 (0.88–6.47)	0.09	0.34 (0.12–0.96)	**0.04**
Over 12	Reference	2.97 (0.8–11.04)	0.1	0.34 (0.09–1.34)	0.12
BMI					
Normal		Reference		Reference	
Overweight	Reference	1.55 (0.75–3.18)	0.24	0.72 (0.33–1.58)	0.42
Obese	Reference	1.24 (0.56–2.73)	0.59	0.55 (0.24–1.26)	0.16
Sex					
Male	Reference	0.54 (0.29–1.01)	0.05	2.38 (1.21–4.67)	**0.01**
ASA class					
ASA 1–2		Reference			
ASA 3–4	Reference	1.01 (0.5–2.04)	0.97	1.28 (0.62–2.65)	0.51
Prior opioid use	Reference	**3.53 (1.73–7.19)**	** < 0.001**	**7.77 (3.69–16.36)**	** < 0.001**
Approach					
Posterior		Reference		Reference	
Anterior	Reference	1.88 (0.47–7.49)	0.37	1.12 (0.21–5.98)	0.9
Circumferential	Reference	1.93 (0.98–3.79)	0.06	0.52 (0.26–1.07)	0.08
Hispanic ethnicity	Reference	5.45 (0.68–43.47)	0.11	0.16 (0.02–1.32)	0.09

Predictors significant at the 0.10 level in univariable analyses were included in a multivariable multinomial logistic regression to predict patient cluster as the response variable. In this model, the Gradual BPS Responders cohort was used as the reference. Coefficients are presented as odds ratios for membership in a particular cluster versus the Gradual BPS Responder cluster, as well as 95% confidence intervals

**Table 4:** Associations between BPS trajectories and long-term outcomes

Postoperative outcomes	Cluster 1: rebound responders	Cluster 2: rapid BPS responders	Cluster 3: gradual BPS responders	*P* value
ED in 30 days	6 (2.33%)	9 (5.11%)	1 (1.75%)	0.25
Readmitted in 30 days	52 (20.16%)	36 (20.45%)	14 (24.56%)	0.21
Hospital LOS in hours (SD)	148.9 (90)	178.5 (128)	101.4 (59)	**0.007**
Postoperative opioid consumption in OME (SD)	679.6 (588)	1008.2 (968)	415.7 (329)	**< 0.001**

Associations between postoperative hospital length of stay (LOS), postoperative opioid use, readmissions, and emergency department (ED) visits were assessed using univariable analysis. Multivariable analyses were subsequently generated for LOS and postoperative opioid use because they were significant at the univariable level. These multivariable models controlled for sex, age, BMI, preoperative opioid use, ASA class, and number of levels fused
